# Analytical Performance of Four Polymerase Chain Reaction (PCR) and Real Time PCR (qPCR) Assays for the Detection of Six *Leishmania* Species DNA in Colombia

**DOI:** 10.3389/fmicb.2017.01907

**Published:** 2017-10-04

**Authors:** Cielo M. León, Marina Muñoz, Carolina Hernández, Martha S. Ayala, Carolina Flórez, Aníbal Teherán, Juan R. Cubides, Juan D. Ramírez

**Affiliations:** ^1^Universidad del Rosario, Facultad de Ciencias Naturales y Matemáticas, Programa de Biología, Grupo de Investigaciones Microbiológicas-UR (GIMUR), Bogotá, Colombia; ^2^Facultad de Medicina, Universidad Nacional de Colombia, Bogotá, Colombia; ^3^Programa de Doctorado en Ciencias Biomédicas y Biológicas, Universidad del Rosario, Bogotá, Colombia; ^4^Grupo de Parasitología, Instituto Nacional de Salud, Bogotá, Colombia; ^5^Residente de Medicina de Emergencias, Escuela de Medicina y Ciencias de la Salud, Universidad del Rosario, Bogotá, Colombia; ^6^Grupo de Investigación COMPLEXUS, Fundación Universitaria Juan N. Corpas, Bogotá, Colombia; ^7^Molecular Biology and Immunology Department, Fundación Instituto de Inmunología de Colombia (FIDIC), Bogotá, Colombia

**Keywords:** *Leishmania*, molecular diagnosis, analytical performance, PCR, qPCR

## Abstract

Leishmaniasis comprises a spectrum of parasitic diseases caused by protozoans of the genus *Leishmania*. Molecular tools have been widely employed for the detection of *Leishmania* due to its high sensitivity and specificity. However, the analytical performance of molecular platforms as PCR and real time PCR (qPCR) including a wide variety of molecular markers has never been evaluated. Herein, the aim was to evaluate the analytical performance of 4 PCR-based assays (designed on four different targets) and applied on conventional and real-time PCR platforms. We evaluated the analytical performance of conventional PCR and real time PCR, determining exclusivity and inclusivity, Anticipated Reportable Range (ARR), limit of detection (LoD) and accuracy using primers directed to kDNA, HSP70, 18S and ITS-1 targets. We observed that the kDNA was the most sensitive but does not meet the criterion of exclusivity. The HSP70 presented a higher LoD in conventional PCR and qPCR in comparison with the other markers (1 × 10^1^ and 1 × 10^-1^ equivalent parasites/mL respectively) and had a higher coefficient of variation in qPCR. No statistically significant differences were found between the days of the test with the four molecular markers. The present study revealed that the 18S marker presented the best performance in terms of analytical sensitivity and specificity for the qPCR in the species tested (species circulating in Colombia). Therefore, we recommend to explore the analytical and diagnostic performance in future studies using a broader number of species across America.

## Introduction

Leishmaniasis comprises a spectrum of diseases caused by a single-celled flagellate protozoan of the genus *Leishmania* and transmitted by the bite of a female phlebotomine of the family Psychodidae (Akhoundi et al., 2016). About 20 species of *Leishmania* are responsible for a wide range of clinical manifestations in humans and vertebrates. Three clinical manifestations exist: (i) cutaneous leishmaniasis (CL) that causes skin lesions, (ii) mucocutaneous leishmaniasis (MCL) characterized by localized mucosal lesions and (iii) visceral leishmaniasis (VL), which is responsible for a severe chronic infection of the reticuloendothelial system that leads to death if it is not treated timely (Bezerra-Vasconcelos et al., 2011). CL is the most common form, characterized by the presence of ulcerative lesions leading to disfiguring and/or incapacitating scars (Desjeux, 2001; Alvar et al., 2012). This neglected tropical disease is considered endemic in large tropical, subtropical and Mediterranean basins (Jara et al., 2013; Akhoundi et al., 2017).

According to data reported by the World Health Organization (WHO), it is prevalent over 98 countries. It is estimated that ∼ 350 million people are at risk of infection and ∼ 12 million cases with an annual (estimated) incidence of 0.7–1.2 million cases of CL and 0.2–0.4 million of VL (Alvar et al., 2012; Pan American Health Organization, [WHO], 2013) are reported. For the old world, CL is most commonly associated with *Leishmania major, L. tropica*, and *L. aethiopica* species. For the Americas, localized CL is caused by multiple species of the subgenus *Leishmania* and *Viannia* (*L. mexicana, L. braziliensis, L. panamensis, L. amazonensis, L. colombiensis, L. guyanensis, L. peruviana* among others). MCL is most frequently associated with *L. braziliensis* and *L. panamensis* species (Pan American Health Organization, [WHO], 2013). The control of leishmaniasis is complicated by the variety of *Leishmania* species and the different clinical forms as well as by unique epidemiological patterns of the disease. In many regions of the New World, two or more species are often sympatric (Hashiguchi et al., 2017), for example, Colombia is the country with the largest number of *Leishmania* species that affect humans in the world and complicates the control of this pathology (nine species in total) (Ramirez et al., 2016).

Traditionally, microscopic examination is considered the diagnostic routine method for CL and MCL. However, despite its high specificity (100%), its sensitivity is low. Several authors report that the sensitivity of direct microscopic examination varies between 74.4 and 40%, and these values depend on aspects related to the evolution of the skin lessions, the localization where the sample is taken and the expertise of the microscopist (Bensoussan et al., 2006; Szargiki et al., 2009; Goto and Lauletta Lindoso, 2012). Therefore, more sensitive methods such as the polymerase chain reaction (PCR) have been developed as an alternative for the diagnosis and identification of *Leishmania* species. PCR platforms show sensitivity values between 92 and 100% and specificity of 100% (Reithinger and Dujardin, 2007; Shahbazi et al., 2008; Mohammadiha et al., 2013; Adams et al., 2014; Munoz et al., 2016). For the amplification of DNA fragments of *Leishmania* species, the use of genetic targets such as kinetoplast DNA (kDNA), which has a sensitivity of 97% and a specificity of 87%, has been reported in several studies (Marques et al., 2001; Rodriguez et al., 2002; Jara et al., 2013). The Heat Shock Protein 70 kDa (HSP70) also reflects a sensitivity of 95% and a specificity of 100% (Garcia et al., 2007; Montalvo et al., 2014).

The internal transcribed spacer 1 (ITS-1) with 40 and 96% respectively (Marfurt et al., 2003; Kumar et al., 2007; Ovalle Bracho et al., 2007) and finally the small 18S ribosomal subunit with a similar behavior as kDNA in terms of sensitivity and specificity (Adams et al., 2014). These are the most commonly used markers for the identification of *Leishmania* DNA by PCR and Real Time PCR. However, these studies only report aspects related to the sensitivity and specificity of the technique. Also, the published studies only evaluate maximum two markers per technique/platform and an adequate evaluation of the analytical performance of the technique and genetic targets employed has never been conducted. This is of critical relevance, because those aspects are mandatory to determine the analytical specificity and sensitivity of molecular methods (Berzunza-Cruz et al., 2002; Marfurt et al., 2003; Chargui et al., 2005; Bensoussan et al., 2006; Ovalle Bracho et al., 2007; Adams et al., 2014; Mouttaki et al., 2014).

The evaluation of the analytical performance is understood as the stage where reproducibility, inclusivity, exclusivity, accuracy and the limit of detection (LoD) are determined as the initial phase for the complete validation of a diagnostic method (NCCLS, 2004; Burd, 2010). Aspects already evaluated in other parasitic diseases such as Chagas disease and Toxoplasmosis (Sterkers et al., 2010; Ramirez et al., 2015). Due to the broad spectrum of leishmaniasis it is mandatory to find a highly sensitive method for diagnosis especially in endemic regions and in the New World where several species co-exist and cause CL (Pourmohammadi et al., 2010). To date, however, the lack of information on the evaluation of the analytical performance of the molecular diagnosis of CL has led to a biased use of PCR and qPCR with several molecular targets. Therefore, the purpose of the study was to evaluate the analytical performance of 4 PCR-based assays (designed on 4 different targets) and applied on conventional and real-time PCR platforms to detect the main New World *Leishmania* species causing CL in Colombia.

## Materials and Methods

### Ethics Statement

This project has a certificate of approval from the ethics committee of the National University of Colombia number 002-010-15 issued on February 12, 2015. This research study did not include samples from humans, animals or any individual.

### Reference *Leishmania* Strains

Promastigote cultures of the major *Leishmania* reference strains frequently associated with CL and MCL in Colombia were donated by the International Center for Medical Research and Training (CIDEIM) that has already-existing collection of *Leishmania* [MHOM/BR/75/M2903 *L. braziliensis*, MHOM/PA/71/LS94 *L. panamensis*, MHOM/BR/75/M4147 *L. guyanensis*, MHOM/TN/80/IPT1 *L. infantum*, IFLA/BR/67/PH8 *L. amazonensis* and MHOM/BZ/82/BEL21 *L. mexicana*]. These strains were cloned and maintained in Novy, Nicolle and McNeal medium and Schneider medium supplemented with 20% fetal bovine serum (Microgen). There was no calculation of sample size due to the lack of availability of the 20 *Leishmania* species that infect humans. We decided to include six from the nine species of *Leishmania* that have been reported in Colombia.

### DNA Extraction and Serial Dilutions

DNA extraction was performed according to the instructions of the High Pure PCR Template Preparation kit (Roche^®^ Ref. 11796828001) from a stock that contained 10^5^ parasite equivalents/mL. DNA obtained from each reference strain was subsequently used to perform serial dilutions from 1 × 10^4^ to 1 × 10^-2^ parasites equivalents/mL to determine the analytical performance of molecular tests (PCR and qPCR).

### Selection of Molecular Targets

To determine the analytical performance of the PCR and qPCR, four of the molecular markers commonly employed in the literature for molecular diagnosis were selected: (i) The gene coding for the heat shock protein of 70 kDa (HSP-70) (Garcia et al., 2007; Cruz et al., 2013); (ii) The ITS-1 (el Tai et al., 2000; Bensoussan et al., 2006; Eroglu et al., 2014; Hernández et al., 2014); (iii) The kinetoplast conserved region (kDNA) (Motazedian et al., 2002; Mary et al., 2004; Boggild et al., 2010; Jara et al., 2013) and (iv) The 18S ribosomal RNA (18S) (Deborggraeve et al., 2008; Bezerra-Vasconcelos et al., 2011; Cruz et al., 2013; Adams et al., 2014). These assays were conducted using primers previously reported (Supplementary Table S1) (Cruz et al., 2002; Medeiros et al., 2008).

### Molecular Tests (PCR and qPCR)

#### Polymerase Chain Reaction

The master mix (one per molecular target) was performed at a final volume of 15 μL which contained 1.5 μL of reaction buffer 10X (Invitrogen), 0.125 μL of dNTPs (10 mM), 0.365 μL of MgCl2 (25 mM), 0.5 μL of each primer (HSP70f and HSP70r; KDNAf and kDNAr, LITS and L5.8S and R223 and R333) (10 μM), 0.05 μL of Taq platinum DNA polymerase (Invitrogen) (0.1U) and 5 μL of DNA. PCR was performed on T100^TM^ Thermal Cycler (Bio-Rad) using thermal cycle conditions as follows: An initial denaturation of 95°C for 5 min followed by 40 cycles at 95°C for 1 min, 1 min at 60°C and 1 min at 72°C, with a final extension at 72°C per 10 min (For the LITS and L5.8S primers, the annealing temperature was 56°C). To determine the band size, the amplification products were run on 2% agarose gels and stained with Sybr Safe.

#### Real Time Polymerase Chain Reaction (qPCR)

The master mix (one per molecular target) was performed at a final volume of 12 μL which contained 5.0 μL of Fast SYBR Green (Applied Biosystems Ref. 4385370), 0.6 μL of each of the same sets of primers used in PCR (shown above) and 2 μL of DNA. The thermal profile consisted of the first stage of 50°C for 2 min followed by 40 cycles at 95°C for 30 s and 15 s at 60°C. The qPCR was executed with a 7500 Fast Real-Time PCR System (Applied Biosystems). After the qPCR assay, a melting curve analysis was performed to detect any primer dimerization that could affect the efficiency of the assays.

### Analytical Specificity

Analytical specificity was evaluated in terms of selectivity, given the test’s responsiveness to selectively identify blank (*Leishmania* DNA) and non-blank (non-*Leishmania* DNA) sample sources. This feature includes:

#### Inclusivity

Describes the ability of the tests to detect the existing diversity of blank DNA (*Leishmania* DNA). Therefore, we performed standard PCR and qPCR with the four molecular markers (HSP70, ITS-1, 18S and kDNA) with the conditions described above to all the DNAs of the six *Leishmania* reference strains within a single day.

#### Exclusivity

Determines the non-response of DNA tests from closely related but not considered target sample sources. In this case, we selected microorganisms phylogenetically related to *Leishmania* and also those associated with differential diagnosis of CL. DNAs of parasites belonging to the order Kinetoplastida and obtained from a biological supply vendor (ATCC: The Global Bioresource Center) (ATCC PRA-330 *Trypanosoma cruzi* and ATCC 30032 *Trypanosoma rangeli*) and 8 microorganisms of differential diagnosis of CL (ATCC 25923 *Staphylococcus aureus*, ATCC 12344 *Streptococcus pyogenes*, ATCC 26033 *Histoplasma capsulatum*, ATCC 27294 *Mycobacterium tuberculosis*, ATCC 26329 *Sporothrix schenckii* and ATCC 18827 *Fonsecaea pedrosoi*) were subjected to conventional PCR and qPCR within a single day.

### Analytical Sensitivity

The analysis of the analytical selectivity of the tests was directed to evaluate the measurement of error that can exist within specified limits. This feature includes:

#### Anticipated Reportable Range (ARR)

It refers to a range of concentrations in which the analyte can be determined with an adequate level of confidence and accuracy. To achieve this, seven serial dilutions (1 × 10^4^ to 1 × 10^-2^ parasites equivalents/mL) of each DNA of the *Leishmania* species (six species) were taken and subjected to the two molecular platforms (PCR and qPCR) with each of the four genetic targets (HSP70, kDNA, 18S and ITS-1). Each dilution was amplified in triplicate within a single day. For the case of conventional PCR, the minimum dilution to which a positive result (present of amplification band in electrophoresis) was consistently generated was identified. For qPCR, the reaction efficiency was evaluated through linear regression analysis, by calculating: (i) the slope of the linear logarithmic phase of the reaction, representing the accuracy and reproducibility of the results (values between -3.0 and -3,6, with -3.32 considered the expected value, corresponding to 100% efficiency), (ii) Y-intercept, corresponding to the theoretical detection limit of the reaction and (iii) correlation coefficient (*R*^2^), as a measure of linearity of the obtained curves and reflection of the reproducibility. Amplification efficiencies for the qPCR were graphically represented using the program GraphPad Prism 7.

#### Limit of Detection (LoD)

The LoD was calculated as the lowest dilution providing 95% positive results, as established by NCCLS standards (NCCLS, 2004). Five serial dilutions of each DNA of the six *Leishmania* species were used and subjected to both molecular platforms with each of the four genetic targets. The amplification of each dilution was performed with 8 replicates and during 5 consecutive days. The LoD was determined by Probit Regression (Probit Minitab 15 software, United States).

#### Accuracy

Intra-assay reproducibility was assessed in terms of accuracy for each test. A dilution above and below the LoD of each DNA of the 6 *Leishmania* species were evaluated in triplicate for 10 days (one run per day) under the same conditions. For qPCR: Mean, Standard Deviation (SD) and Coefficient of Variation (CV) were estimated. For PCR, the presence/absence of a band was considered as a result for the subsequent analyses.

### Comparative Statistical Analysis

The reproducibility of the qPCR results was evaluated through an initial analysis of variance homogeneity (based on the F2 distribution), followed by the implementation of comparison tests of means (considering the same or different variances, as the case may be). This set of analysis was aimed at comparing the means of Ct and their corresponding SD at three levels: (i) days, (ii) molecular marker and iii) evaluated species. A value of *p* < 0.05 was considered statistically significant for this set of hypothesis tests.

The variation of the results depending on the concentration of the blank DNA (from each *Leishmania* species) was compared among the tests through descriptive analyzes. For conventional PCR the amplification’s minimum dilution was determined by identifying the dilution in those results were consistently positive (>90%) considering the total of developed trials: ARR (n: 3): 3 replicates in a single day; LoD (n: 40): 8 replicates during 5 days; Accuracy (n: 30): 3 replicates during 10 days. The dispersion measures (standard deviation ‘SD’) were calculated considering the positive result per total trials carried out per dilutions. For the qPCR the percentage of CV for each dilution was considered with respect to the maximum CV. The comparison between tests was carried out through a graphical representation, assigning one color per range of variation (every 20%).

A one-way ANOVA test was used to explore relationships between species, target and parasite concentration with mean Ct, and a Bonferroni-corrected factorial ANOVA (*Post Hoc*) to determine interactions between variables and to identify the most influential subcategories in the average Ct. Likewise, the interaction between these three variables was determined and a *p*-value < 0.01 was established as significant.

## Results

### Inclusivity and Exclusivity for PCR and qPCR

The tests of amplification from the DNAs of the 6 *Leishmania* species showed bands in the expected sizes for PCR and emission of fluorescence by qPCR across all the samples. When we retrieved the results from primer dimerization in the melting curve analysis, we only detected one peak excluding the previous premise. We concluded that both tests are inclusive. In terms of exclusivity, we observed that the test directed to kDNA was not exclusive in both platforms (PCR and qPCR) for the amplification of *Leishmania*, because we detected amplification with *T. cruzi* and *M. tuberculosis* DNAs (**Supplementary Figure S1**).

### ARR, LoD and Accuracy for PCR

**FIGURE 1:**
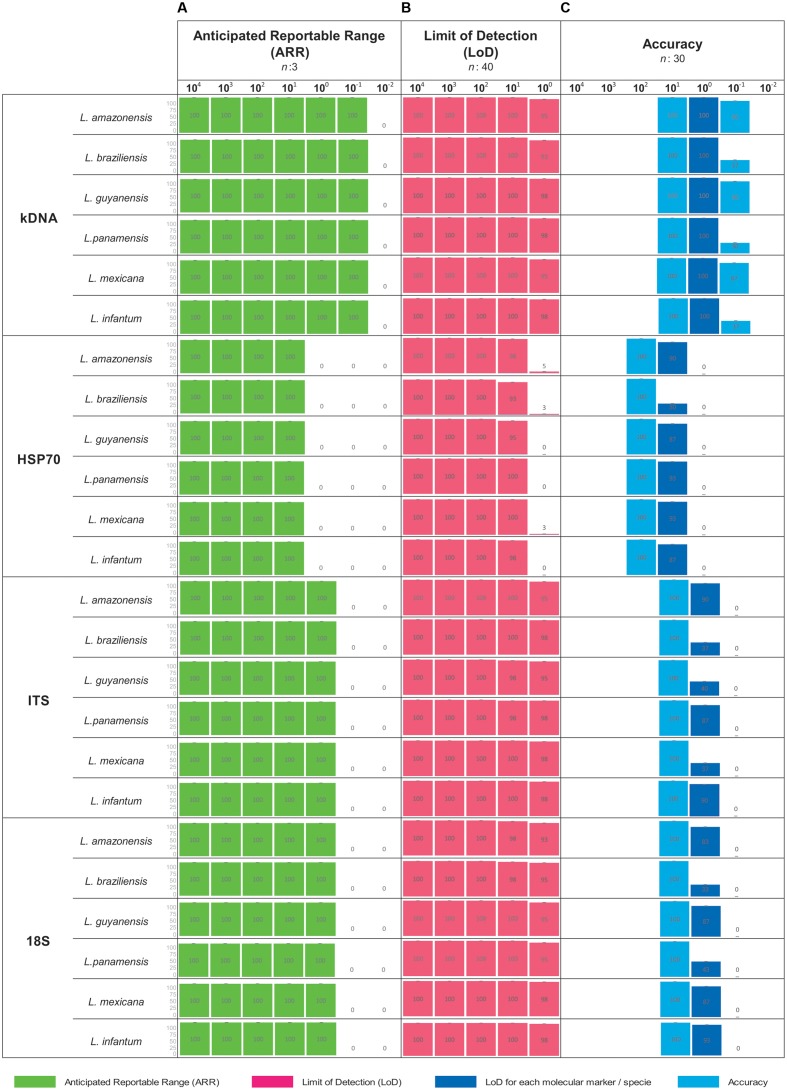
Analytical sensitivity for conventional PCR. For each concentration a result is determined as positive when band presence was observed. **(A)** ARR determined from 7 serial dilutions analyzed for the four markers employed across the seven species studied; **(B)** LoD as a consensus of 5 serial dilutions for the four markers employed across the seven species studied and **(C)** Accuracy. Including dilution above and below the LoD for the four markers employed across the seven species studied.

Consensus results were obtained from each dilution in each parameter evaluated (ARR, LoD and accuracy). The broadest ARR was observed with the kDNA marker obtaining amplification up to the 1 × 10^-1^ dilution for all *Leishmania* species. For the ITS-1 and 18S markers the ARR is reported from the dilutions 1 × 10^4^ to 1 × 10^0^ and finally the marker HSP70 reports a low amplification range (1 × 10^1^ parasite equivalents/mL) (**Figure 1A**). It was determined that the LoD for the markers kDNA, ITS-1 and 18S was up to the dilution 1x10^0^ parasites equivalents/mL. For the HSP70 marker the LoD was 1x10^1^ parasites equivalents/mL for all *Leishmania* species (**Figure 1B**). These results are consistent with the ARR. As for the accuracy of the PCR during the 10 days of analysis, we concluded that it is low, neither homogeneous nor accurate data were observed as obtained in the ARR and LoD. In some days no amplification was obtained in the LoD (**Figure 1C**).

### ARR for qPCR

**FIGURE 2:**
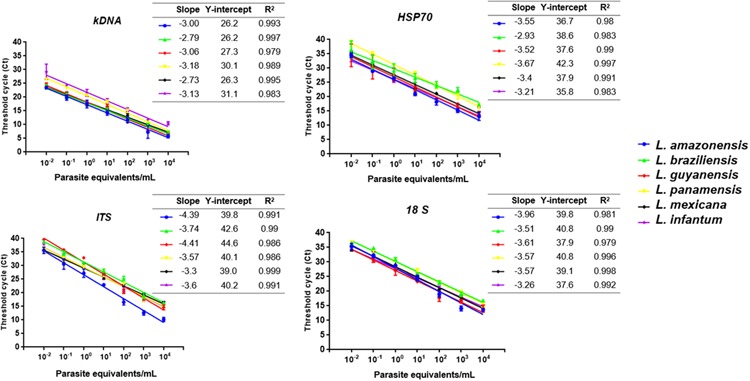
Linear regression results for each of the four molecular marker employed with its respective *Leishmania* species including (i) the slope of the linear logarithmic phase of the reaction, representing the accuracy and reproducibility of the results (values between -3.0 and -3.6, with -3.32 considered the expected value, corresponding to 100% efficiency), (ii) Y-intercept, corresponding to the theoretical detection limit of the reaction and (iii) correlation coefficient (*R*^2^).

The linear regression results for each molecular marker with its respective *Leishmania* species is shown in **Figure 2**. For the kDNA was observed that the amplification covers all dilutions of the ARR at a very early Ct (about 5–29). The values of slopes obtained for *L. mexicana* (-2.73) and *L. braziliensis* (-2.79) did not meet the expected values (**Figure 2**). In general, the technique presents good efficiency and reproducibility among replicates in *L. amazonensis, L. guyanensis, L. panamensis* and *L. infantum.* For the HSP70 marker, the slope values for *L. braziliensis* (-2.93) were not within the range but the technique presents good efficiency and reproducibility between replicates in the other species. A good reproducibility of the technique with the ITS-1 marker was determined but a low efficiency in the *L. amazonensis* (-4.39) and *L. guyanensis* (-4.41) species. Finally, 18S showed a very homogeneous ARR among all species compared to kDNA, HSP70 and ITS-1 (good reproducibility and efficiency of the technique) (**Figure 2**).

### LoD and Accuracy of qPCR

**FIGURE 3:**
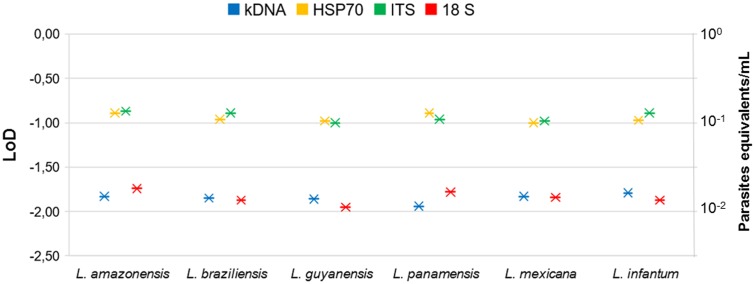
Probit regression to determine LoD of qPCR for the four molecular markers and six *Leishmania* species.

To determine LoD of the qPCR platform, we first determined whether there was variation on the day of the tests. With the data presented in **Supplementary Figure S2**, a comparison test of variances with a 95% confidence interval was performed. We observed that no statistically significant differences were found between the days with the 4 molecular markers in the 5 serial dilutions (*P*-value: 0.86). A Probit regression analysis was then performed (**Figure 3**). The LoD for kDNA and 18S in qPCR was 1 × 10^-2^ parasites equivalents/mL and for HSP70 and ITS-1 was 1 × 10^-1^ parasites equivalents/mL. The complete results of Probit regression can be observed in Supplementary Table S2. Regarding the accuracy, **Table 1** compare the means, SD and accuracy of the 6 species of *Leishmania* in each of the four molecular markers.

**Table 1:** Comparison of the LoD in terms of means, standard deviation, coefficient of variation percentage as a measure of accuracy of the 6 species of *Leishmania* for each molecular marker by qPCR.

Marker	Species	10^-3^ (0,001 parasite equivalents/mL)			10^-2^ (0.01 parasite equivalents/mL)			10^-1^ (0.1) parasite equivalents/mL			10^0^ (1 parasite equivalent/mL)		
					
		Mean (Ct)	*SD*	CV%	Mean (Ct)	*SD*	CV%	Mean (Ct)	*SD*	CV%	Mean (Ct)	*SD*	CV%
HSP70	*L. amazonensis*				33.32	1.76	5.29	29.87	0.95	3.19	27.45	0.37	1.34
	*L. braziliensis*				33.29	1.60	4.82	30.09	0.74	2.44	27.56	0.47	1.69
	*L. guyanensis*				31.88	1.79	5.60	29.92	1.01	3.36	27.5	0.52	1.88
	*L. panamensis*				31.75	2.66	8.37	30.26	0.86	2.83	27.59	0.43	1.55
	*L. mexicana*				33.99	1.46	4.29	30.38	0.68	2.24	28.05	0.48	1.71
	*L. infantum*				34.29	2.19	6.39	30.03	0.63	2.10	27.43	0.45	1.66

ITS	*L. amazonensis*				36.76	0.84	2.30	34	0.37	1.07	30.14	0.37	1.24
	*L. braziliensis*				36.56	0.80	2.19	34.22	0.39	1.15	30.07	0.33	1.09
	*L. guyanensis*				37.58	0.85	2.26	34.43	0.39	1.13	30.14	0.35	1.17
	*L. panamensis*				37.14	0.79	2.13	33.93	0.41	1.21	30.03	0.47	1.57
	*L. mexicana*				37.55	0.65	1.74	34.48	0.39	1.14	30.21	0.44	1.46
	*L. infantum*				36.77	0.75	2.03	34.38	0.43	1.26	29.98	0.36	1.21

kDNA	*L. amazonensis*	29.57	0.34	1.14	23.83	0.24	0.99	20.3	0.22	1.07			
	*L. braziliensis*	29.85	0.31	1.04	24.52	0.11	0.46	19.46	0.20	1.00			
	*L. guyanensis*	30.1	0.37	1.24	24.67	0.34	1.39	19.44	0.26	1.32			
	*L. panamensis*	29.99	0.45	1.49	24.54	0.26	1.09	20.18	0.21	1.05			
	*L. mexicana*	30.41	0.40	1.33	24.09	0.30	1.24	20.21	0.22	1.11			
	*L. infantum*	29.99	0.40	1.35	24.09	0.31	1.27	19.99	0.21	1.06			

18 S	*L. amazonensis*	39.03	0.63	1.61	34.91	0.45	1.30	31.51	0.43	1.35			
	*L. braziliensis*	38.74	0.56	1.44	35.03	0.37	1.05	31.27	0.39	1.24			
	*L. guyanensis*	38.89	0.78	2.01	34.83	0.46	1.33	31.25	0.58	1.84			
	*L. panamensis*	39.06	0.49	1.24	34.72	0.41	1.17	31.44	0.45	1.44			
	*L. mexicana*	38.67	0.81	2.10	34.77	0.61	1.76	31.32	0.40	1.28			
	*L. infantum*	39.62	0.76	1.92	34.75	0.47	1.36	31.53	0.48	1.51			


### Comparison of CV for PCR vs. qPCR

**FIGURE 4:**
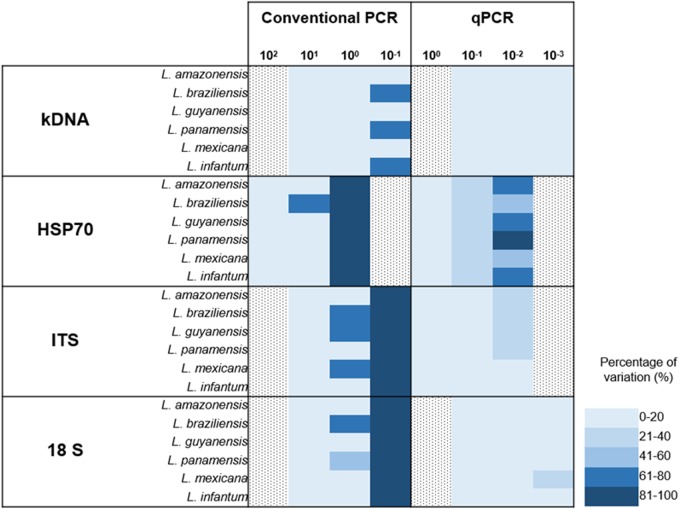
Absolute comparison of percentages of variation in conventional and real time PCR for the four markers employed across the seven species studied.

Finally, a graphical representation of the percentage of variation of the two techniques was constructed (PCR vs. qPCR). Here, we identified that at very low dilutions the coefficient of variation between replicates increases (**Figure 4**). Percentages of variation of up to 100% were identified in markers such as ITS-1 and 18S in dilutions of 10^-1^ parasite equivalents/mL in conventional PCR. However, in the case of qPCR, kDNA and 18S showed reduced percentages of variation, even when the test is performed to lower dilutions.

### Comparative Statistical Analyses

A total of 2086 trials were performed to evaluate the accuracy, 4795 for the LoD and 500 for the ARR. Respectively, 74 (3.5%), 5 (0.1%), and 4 (0.8%) trials where Ct was undetectable were presented. The Ct (SD) averages in the assessments of accuracy, LoD and ARR were 30.7 (0.11), 19.4 (6.5), 22.7 (8.31), respectively.

In the three evaluations, the univariate analyzes showed a relation between the average Ct, the targets and concentrations (*p*: 0000). In the evaluation of the LoD, although with very low *R*^2^ (4.44%), it was identified relationship between the species and the average Ct (p: 0000). This relationship between the average Ct and the species was not identified in the other two assessments (data not shown). Interaction between the three factors was identified, except for the evaluation of the ARR, where only a bivariate type relationship was present, but not among the three factors (**Table 2**).

**Table 2:** Results of the Factorial ANOVA – Threshold Cycle (Ct) to determine multivariate interactions among target, species and concentration in the ARR, LoD and Accuracy parameters.

Condition	df	*F*	*p*-value
**ARR**			
Target (T)	3	2108.158	0.000
Species (S)	5	113.246	0.000
Concentration (C)	6	3170.955	0.000
T^∗^S	15	31.046	0.000
T^∗^C	18	10.043	0.000
S^∗^C	30	4.902	0.000
S^∗^C^∗^T	90	1.781	0.014
**LoD**			
Target (T)	3	13893.279	0.000
Species (S)	5	1212.73	0.000
Concentration (C)	4	18733.319	0.000
T^∗^S	15	192.626	0.000
T^∗^C	12	79.141	0.000
S^∗^C	20	47.799	0.000
S^∗^C^∗^T	60	17.816	0.000
**Accuracy**			
Target (T)	5	3.046	0.000
Species (S)	3	9735.668	0.000
Concentration (C)	3	4317.608	0.000
T^∗^S	15	3.965	0.000
T^∗^C	15	1.171	0.000
S^∗^C	5	68.932	0.000
S^∗^C^∗^T	25	4.116	0.000


The mean Ct decreased as the parasite concentration increased (*p*: 0.000). On the other hand, the target that was related to an average Ct lower was the kDNA, and in the *post hoc* analysis, differences were always found in the paired evaluation of the average Ct of the targets (*p*: 0.000), except for HSP-70 with 18S, specifically in the LoD and ARR (Supplementar Table S3).

## Discussion

In the literature, the detection of *Leishmania* has been reported by PCR due to its high sensitivity in comparison with traditional parasitological methods (Kumar et al., 2007) and their ability to detect DNA of the parasite in a wide variety of clinical specimens (skin biopsy, ulcer material, blood, bone, bone marrow, lymph nodes, and direct smears) (Pérez et al., 2011; Mohammadiha et al., 2013). For the molecular diagnosis, several primers directed to genetic targets have been evaluated including ITS-1, kDNA, HSP70, SSUrRNA, Miniexon among others. These studies only report the operational capabilities of the assays for unique *Leishmania* species. Nevertheless, an adequate evaluation of the analytical performance of the techniques and primers directed to the genetic targets has not been yet performed and still mandatory (Berzunza-Cruz et al., 2002; Marfurt et al., 2003; Chargui et al., 2005; Bensoussan et al., 2006; Garcia et al., 2007; Kumar et al., 2007; Ovalle Bracho et al., 2007; Deborggraeve et al., 2008; Al-Hucheimi et al., 2009; Mouttaki et al., 2014; Akhoundi et al., 2017). Only certain studies have evaluated parameters of the technique’s exclusivity (Salotra et al., 2001), evaluation of ARR (Jara et al., 2013) and determination of LoD (Santamaria et al., 2005; Deborggraeve et al., 2008; Hernández et al., 2014; Hitakarun et al., 2014). Cruz et al. (2013) report the unique multi-center study on the evaluation of diagnostic tools for leishmaniasis. They report the sensitivity of the different molecular methods in four laboratories in endemic areas, concluding that the qPCR with the kDNA gene presented the highest sensitivity, whereas the qPCR directed to the ITS-1 and the digestion with the enzyme HaeIII plus the HSP70 + RLFP combination were the most appropriate targets for species identification (Cruz et al., 2013). Herein, we evaluated for the first time to our knowledge the analytical performance of PCR and qPCR assays using previously reported primers directed to 4 genetic targets in six endemic and causative CL species from the New World. However, it is well known the vast diversity across *Leishmania* species in the Americas. This is a limitation of our study and further investigations should consider more species.

### Analytical Specificity

Regarding the exclusivity of the technique with the four markers, we observed that the PCR assay herein tested is not exclusive for *Leishmania* DNA amplification when using the primers selected and directed to kDNA, due to the amplification with DNA of *T. cruzi* and *M. tuberculosis* (**Supplementary Figure S1**). One explaination might be the use of degenerated primers despite of the good performance of these primers in the initial *in silico* evaluation. These findings contrasts the reported by Salotra et al. (2001), where there was no cross-reaction with *M. tuberculosis* and *M. leprae* DNA (Salotra et al., 2001). This has to be considered since there are several reported primers directed to the conserved and hypervariable regions of *Leishmania* kDNA that do not show cross-reaction with *T. cruzi* DNA (Gualda et al., 2015; Ceccarelli et al., 2017). Future studies should implement all the reported primers directed to the kDNA to finally conclude if this marker is or not exclusive for *Leishmania* DNA detection.

The other assays directed to 18S, kDNA and ITS-1 markers were exclusive for *Leishmania* DNA detection, similar to those results reported by Hitakarun et al. (2014). Several studies include in their molecular tests DNA from cross-reactive microorganisms (Salotra et al., 2001; Deborggraeve et al., 2008; Hitakarun et al., 2014). We included eight microorganisms of differential diagnosis with the four assays, corroborating the results obtained in the conventional PCR when using the primers directed to the kDNA. In qPCR, we also detected the DNA amplification of *T. cruzi* possibly for the close phylogenetic relatedness with *Leishmania* (de Morais et al., 2015) and *M. tuberculosis* maybe due to the intraspecies polymorphisms of kDNA as described by Srivastava et al. (2011) and the presence of subclasses across the minicircle molecules (Ceccarelli et al., 2014). Future studies should include the vast variation of kDNA primers to rule out our findings and finally decide if kDNA is advisable or not for the molecular diagnosis of *Leishmania* DNA. However, the primers herein employed were not exclusive. Lastly, no evidence of cross-reaction was depicted with the primers herein directed to ITS-1, HSP70 and 18S.

### Analytical Sensitivity

#### Conventional PCR

We determined the analytical sensitivity for PCR in terms of ARR, LoD and accuracy (**Figure 1** and **Table 1**). Santamaria et al. (2005) determined the LoD in 1 equivalent parasite in the PCR using kDNA (Santamaria et al., 2005), unlike the findings of this study, the LoD of this technique with the same marker was 1 × 10^-1^ parasites equivalents/mL (**Figure 1**). These results are consistent with those described by other authors where is reported that kDNA presents high copy number and can detect up to less than one parasite in conventional PCR (de Bruijn and Barker, 1992; Mary et al., 2004; Francino et al., 2006; Oliveira et al., 2011; Gualda et al., 2015). The LoD for the assays directed to the 18S and ITS-1 markers was 1 × 10^0^ parasites equivalents/mL, similar results reported by Deborggraeve et al. (2008) where the technique was able to detect up to 1 parasite/180 μL. On the other hand, the LoD with the HSP70 marker was 1 × 10^1^ parasites equivalents/mL. These findings support the reported in previous studies where the LoD of HSP70 is lower than other molecular markers. This can be explained by the low number of HSP70 copies across the *Leishmania* genome compared to kDNA and ribosomal markers (Hernández et al., 2014; Hitakarun et al., 2014). Also, this might be explained by the variation across the HSP70 gene in several *Leishmania* species from the old world and new world (Hernández et al., 2014).

When the same parameters were evaluated in qPCR, it was observed that the technique was more sensitive and reproducible than conventional PCR (**Figures 1**, **2**). Previous studies have described these same findings not only for *Leishmania* (Pourmohammadi et al., 2010; Sterkers et al., 2010; Eroglu et al., 2014) but for other microorganisms such as *Helicobacter pylori* (de Bruijn and Barker, 1992), *Plasmodium* spp (Gama et al., 2007), *Salmonella enterica* (Parker et al., 2011) and viruses such as ZIKV (Francino et al., 2006) and DENV (Bai et al., 2008; Faye et al., 2013). For the ARR, we observed that when kDNA is used a Ct between 5 and 27 is observed, a much lower range than the one reported for the other molecular markers (**Figure 2**). According to data reported by Jara et al. (2013), where its amplification range was between a Ct of 7 and 27 (Jara et al., 2013). For the other markers, there was greater variability of the range among strains such as *L. panamensis* and *L. braziliensis* in HSP70 and a low efficiency in *L. amazonensis* and *L. guyanensis* species in ITS-1 (**Figure 2**). In general, good reproducibility and efficiency were presented with kDNA and 18S genetic targets (*R*^2^) between replicates in accordance with Bezerra-Vasconcelos et al. (2011). However, kDNA is not exclusive and the best analytical performance is finally observed for 18S.

The qPCR LoD was 1 × 10^-2^ parasites equivalents/mL for the kDNA and 18S markers, and 1 × 10^-1^ parasites equivalents/mL for HSP70 and ITS-1 (**Figure 3**). These results are consistent with those found by Mary et al. (2004) and Bezerra-Vasconcelos et al. (2011) when using the kDNA, a LoD of 0.0125 parasites/mL was reported but contrasts with Nicolas et al. (2002) where they only report a limit of 0.1 parasites. For the ITS-1 marker, a number of copies from 20 to 200 have been estimated in the *Leishmania* genome and might explain its good performance in low concentrations of parasites (Schonian et al., 2001a,b; Odiwuor et al., 2011). Nevertheless, our results contrast with the reported limit, since the LoD was lower than that found with the 18S gene. For the HSP70, LoD was lower in comparison to kDNA and 18S, but higher than that reported by Hernández et al. (2014), where they report a LoD of 10 parasites/mL (Hernández et al., 2014). For this gene, the number of copies present in the different species, although variable, are few, fluctuating between 1 and 15 copies (MacFarlane et al., 1990; Bock and Langer, 1993; Zurita et al., 2003), which could, theoretically, explain the lowest LoD obtained. It is quite unlikely to estimate the LoD of HSP70 as 1 × 10^-1^ equivalent parasites/mL due to previous reports of 10–15 copies across the genome. However, herein we used a smaller fragment of HSP70 designed exclusively for the New World species. This might have improved the efficiency of the test that allowed us to reach that LoD. This set of primers has never been applied in the old world *Leishmania* species. Also, it is currently unknown the precise organization of the HSP70 gene in the New World species. The advent of Genomic studies will provide further insights about the true number of copies and genomic organizations of HSP70 cluster in new world species. Nevertheless, this is not sufficient to explain the obtained LoD for HSP70. The accuracy results shows that at this dilution (1 × 10^-1^ parasites equivalent/mL), the percentage of variation was 21–40% (**Figure 4**) which was not the case for the other markers demonstrating that there is massive variation at the LoD dilution. This could envisage that small DNA fragments of the gene are subject of amplification via the qPCR but no stable equivalent parasites are truly amplified. Therefore, a plausible explanation might be that the LoD is not stable due to massive variation in terms of gene arrangements or intra-specific variation or enough template availability and plausibly suggesting that in future studies this LoD has to be subject of investigation.

The literature reports that each parasite species contains large numbers of copies of the 18S ribosomal gene (∼ 160). Therefore, several studies choose this marker as the ideal target for molecular studies. In 2011, Bezerra et al., established a LoD of 40 parasites/mL when using 18S. However, Schulz et al. (2003) reported a LoD of 100 parasites/mL. Our results differ from these two studies in determining a lower LoD for 18S (1 × 10^-2^ parasites equivalents/mL). There was no variation in LoD between strains when using this molecular marker (**Table 1**). The data analyzed by ANOVA with three parameters (Variable result: CT, Fixed factors: concentration, species, marker and Covariable: days) had no effect of the covariate nor of the repetition through the days. The same was found when analyzing the difference between the Ct means obtained (*P*-value: 0.86) (**Supplementary Figure S2**). It was also observed that the means comparison tests allowed to identify that the tendency to increase Ct, is dependent on the increase of the dilution, showing a significant difference for all the dilutions and in all the markers (*P*-value < 0.05), When comparing the Ct means of each molecular marker used in each dilution evaluated, it was found that most have a different behavior (statistically significant differences *P*-value < 0.05).

For the accuracy, we did not observe variation between the replicates of the tests, independent of the day of execution of the technique (average coefficient of variation of 0.98), but when handling lower dilutions the coefficient of variation increases in the days of repetition of the technique. This has been demonstrated in studies evaluating the performance of qPCR in *T. cruzi* and in *Leishmania* (Duffy et al., 2013; Jara et al., 2013). The highest coefficient of variation was obtained with the HSP70 marker.

In general, the comparison herein conducted showed the limitations of HSP70 in the detection of *Leishmania* in terms of sensitivity by PCR and qPCR. Also, the fact that kDNA is not an exclusive marker due to the amplification for *T.* cruzi and *M. tuberculosis* DNA (**Supplementary Figure S1**). Also, the low efficiency of the qPCR using the ITS-1 with *L. amazonensi*s and *L. guyanensis* species (**Figure 2**). However, we report the good analytical performance (in terms of accuracy among the species) that the 18S marker exhibits for molecular diagnostics. As mentioned initially, studies on the comprehensive evaluation of the analytical performance of molecular methods for the diagnosis of CL are scarce and are affected by the difficulty of not having a consensus of established diagnostic tests. Implementing a molecular technique such as qPCR for field work in endemic areas for leishmaniasis leads to having a special infrastructure as well as the acquisition of costly equipment and reagents (its cost is reported to be up to three times higher than that of conventional PCR) (Bock and Langer, 1993). This reflects the need to develop new technologies more sensitive but easy to acquire and to be implemented in this type of regions, such as LAMP (Notomi et al., 2000; Tomita et al., 2008; Nzelu et al., 2014, 2016; Abbasi et al., 2016) and nanoparticles (Andreadou et al., 2014). Although microscopy remains the gold standard for routine diagnosis, the high incidence of CL in different regions of South America highlights the need to rethink the implementation of specific strategies for the correct and timely diagnosis of this disease. The results herein obtained provide the basis for the subsequent evaluation of the diagnostic performance with a panel of varied samples and its interlaboratory comparison of real-time PCR with the 18S. We also suggest the inclusion of more species from the Americas. We employed only six species that are the most frequent in CL cases in Colombia but further studies should be considered.

## Author Contributions

CL, CH, and JR conceived and designed the experiments. CL, JC, and JR wrote the manuscript. CL performed the experiments. MM and AT performed statistical analysis. MM, JR, CH, CF, and MA reviewed and revised the manuscript.

## Conflict of Interest Statement

The authors declare that the research was conducted in the absence of any commercial or financial relationships that could be construed as a potential conflict of interest.

## Supplementary Material

**FIGURE S1 |** Conventional PCR amplification in agarose gels (A) kDNA, (B) HSP70, (C) ITS-1, (D) 18S. **Left:** Exclusivity 1: *T. cruzi*; 2: *T. rangeli*; 3: *S. aureus*; 4: *S. pyogenes*; 5: *H. capsulatum*; 6: *M. tuberculosis*; 7: *S. schenckii*; 8: *F. pedrosoi*; 9: positive control; 10: negative control. **Right:** Inclusivity 1: *L. amazonensis*; 2: *L. braziliensis*; 3: *L. guyanensis*; 4: *L. panamesis*; 5: *L. mexicana*; 6: *L. infantum*; 7: Negative control.

**FIGURE S2 |** Differences of the mean Cts for the markers kDNA, HSP70, ITS-1 and 18S.
