# Supplementary figures and images for: Analytical Performance of Four Polymerase Chain Reaction (PCR) and Real Time PCR (qPCR) Assays for the Detection of Six Leishmania Species DNA in Colombia
Source: Front Microbiol. 2017 Oct 4;8:1907. doi: 10.3389/fmicb.2017.01907 (PMC5632848; doi:10.3389/fmicb.2017.01907)

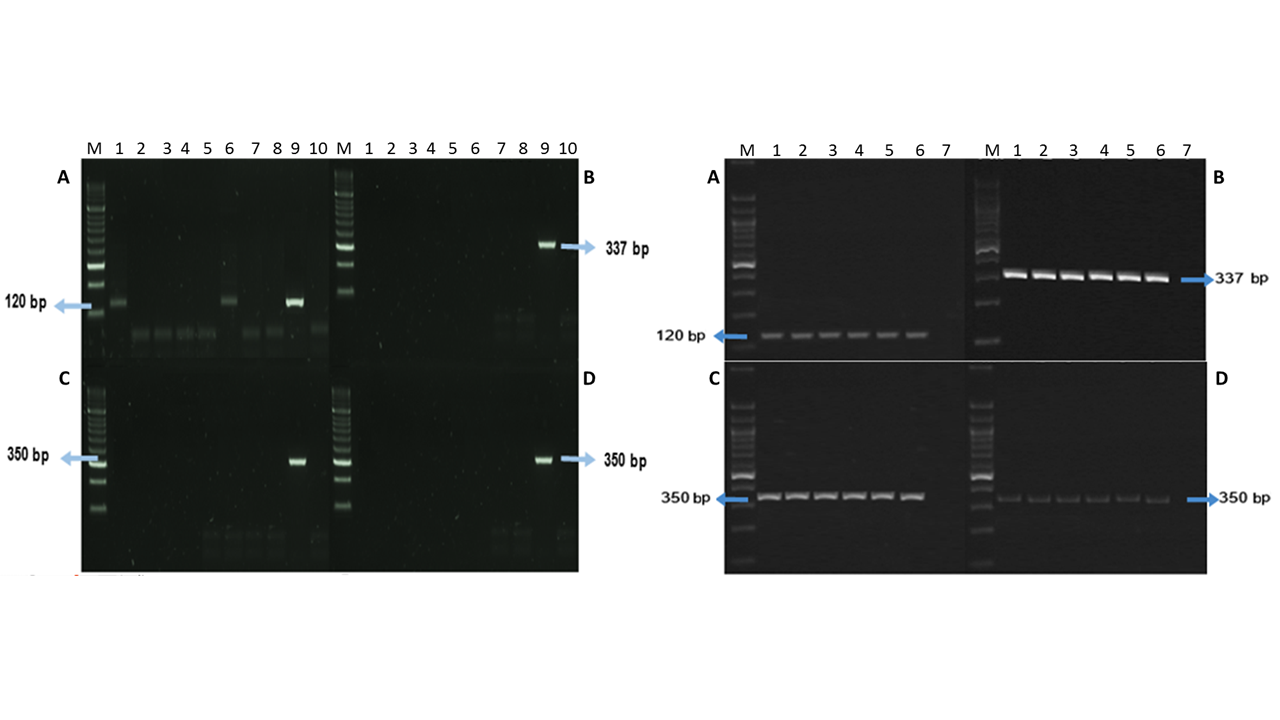

Supplement: Supplementary file 1 [file Image_1.TIF]

## kDNA

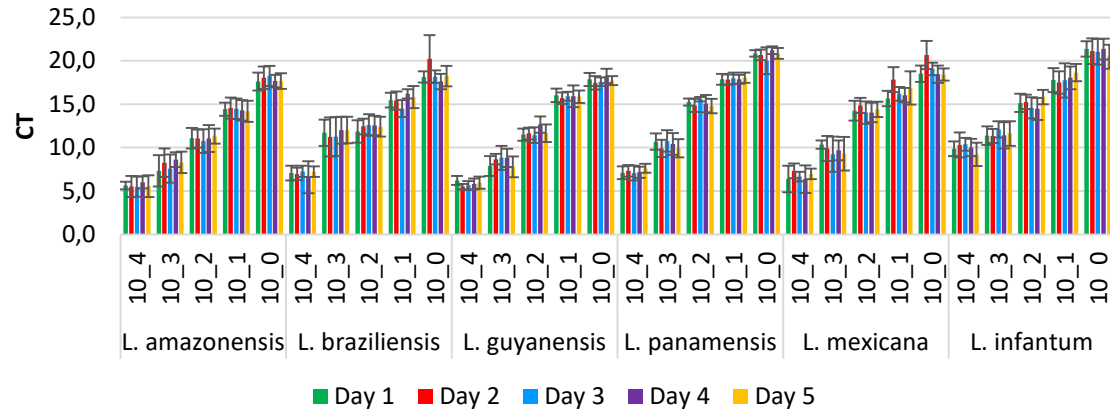

## HSP70

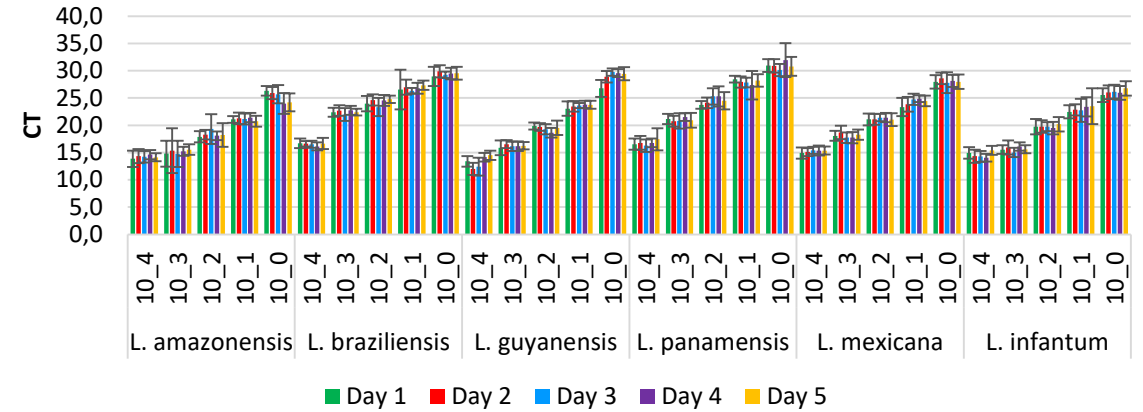

## ITS-1

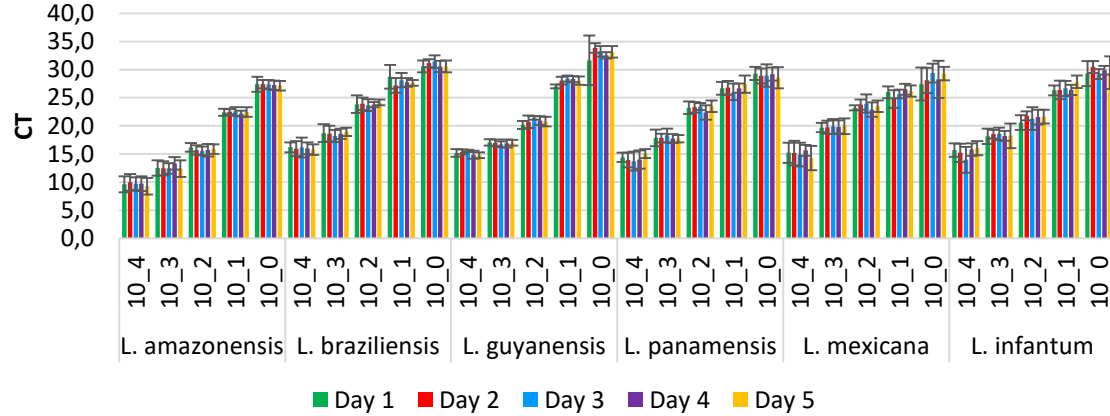

## 18S

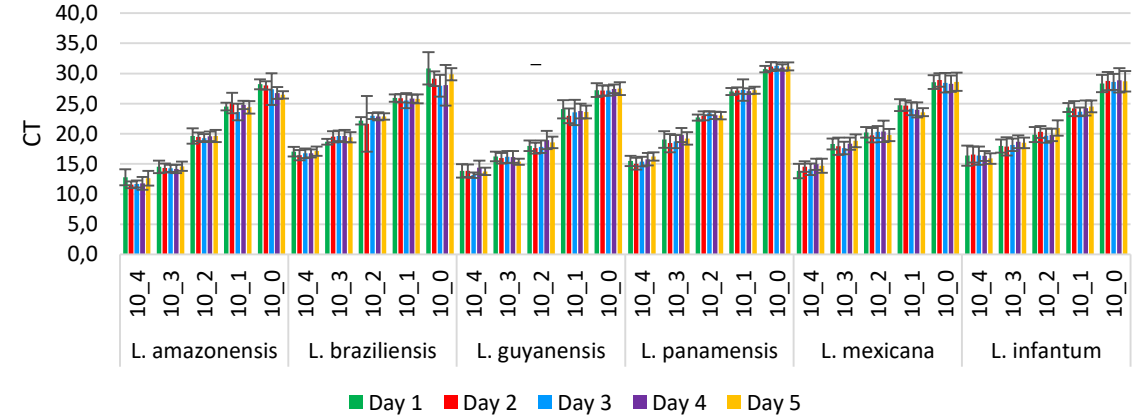

Supplement: Supplementary file 2 [file Image_2.PDF]
