# Supplementary material for: Analytical Performance of Four Polymerase Chain Reaction (PCR) and Real Time PCR (qPCR) Assays for the Detection of Six Leishmania Species DNA in Colombia
Source: Front Microbiol. 2017 Oct 4;8:1907. doi: 10.3389/fmicb.2017.01907 (PMC5632848; doi:10.3389/fmicb.2017.01907)
Supplement: Supplementary file 3 [file Table_1.DOC]

Table S1. Sequences of the primers used in the PCR and qPCR

| **Target** | **Sequence (5´to 3´)** | **Product (bp)** | **Accession number** | **Reference** |
| --- | --- | --- | --- | --- |
| HSP70 Forward | AGGTGAAGGCGACGAACG | 337 bp | AF291716.1 | Hernández et al., 2014 |
| HSP70 Reverse | CGCTTGTCCATCTTTGCGTC |
| ITS – LITSR | CTGGATCATTTTCCGATG | 300 – 350 bp | AJ249612 | El Tai et al ., 2000 |
| ITS - L5.8S | TGATACCACTTATCGCACTT |
| ADNk Forward | (G/C) (G/C) (C/G) CC (A/C) CTA T (A/T) TTA CAC CCA ACC CC | 120 bp | EU429383.1 | Madeiros et al., 2008 |
| ADNk Reverse | GGG GAG GGG CGT TCT GCG AA |
| 18S - R223 | TCCCATCGCAACCTCGGTT | 350 bp | MF000384.1 | Cruz et al., 2002 |
| 18S - R333 | AAAGCGGGCGCGGTGCTG |
