# Supplementary material for: Analytical Performance of Four Polymerase Chain Reaction (PCR) and Real Time PCR (qPCR) Assays for the Detection of Six Leishmania Species DNA in Colombia
Source: Front Microbiol. 2017 Oct 4;8:1907. doi: 10.3389/fmicb.2017.01907 (PMC5632848; doi:10.3389/fmicb.2017.01907)
Supplement: Supplementary file 4 [file Table_2.DOC]

**Table S2.** Limit of Detection determined by regression Probit Analysis

| **Target** | ***Species*** | **L**o**D** | **Parasite equivalents/mL** | ***Y Intercept (Ct) [CI 95%]*** | ***F*** | ***p*** |
| --- | --- | --- | --- | --- | --- | --- |
|  |  |  |  |  |  |  |
| **kDNA** | *L. amazonensis* | -1,83 | 1 x 10^-2^ | 26,3 [24,8 - 27,9] | 422 | 0,00025 |
|  | *L. braziliensis* | -1,85 | 1 x 10^-2^ | 26,4 [25 - 27,7] | 449,5 | 0,00022 |
|  | *L. guyanensis* | -1,86 | 1 x 10^-2^ | 27,2 [25,2 - 29,1] | 277,2 | 0,0004 |
|  | *L. panamensis* | -1,94 | 1 x 10^-2^ | 27,2 [25,5 - 28,6] | 342,7 | 0,0002 |
|  | *L. mexicana* | -1,83 | 1 x 10^-2^ | 26,7 [25,3 - 28,1] | 393,7 | 0,00027 |
|  | *L. infantum* | -1,79 | 1 x 10^-2^ | 27,3 [25,9 - 28,7] | 223,7 | 0,0001 |
| **HSP70** | *L. amazonensis* | -0,89 | 1 x 10^-1^ | 30,4 [29,5 - 31,3] | 789,2 | 0,00009 |
|  | *L. braziliensis* | -0,96 | 1 x 10^-1^ | 31,3 [30,4 - 32,2] | 649,4 | 0,00006 |
|  | *L. guyanensis* | -0,98 | 1 x 10^-1^ | 30,8 [29,9 - 31,7] | 724,5 | 0,00006 |
|  | *L. panamensis* | -0,89 | 1 x 10^-1^ | 29,8 [28,9 - 30,7] | 764,4 | 0,00007 |
|  | *L. mexicana* | -1,0 | 1 x 10^-1^ | 30,2 [29,3 - 31,1] | 689,3 | 0,00007 |
|  | *L. infantum* | -0,97 | 1 x 10^-1^ | 30,4 [29,5 - 31,3] | 783,3 | 0,00008 |
| **ITS-1** | *L. amazonensis* | -0,87 | 1 x 10^-1^ | 35,4 [34 - 37,1] | 567,4 | 0,00008 |
|  | *L. braziliensis* | -0,89 | 1 x 10^-1^ | 34,6 [33,2 - 36,3] | 474,6 | 0,0002 |
|  | *L. guyanensis* | -1,0 | 1 x 10^-1^ | 34,4 [33 - 36,1] | 510,1 | 0,0002 |
|  | *L. panamensis* | -0,96 | 1 x 10^-1^ | 34,7 [33,3 - 36,4] | 420,3 | 0,0004 |
|  | *L. mexicana* | -0,98 | 1 x 10^-1^ | 34,5 [33,1 - 36,2] | 484,6 | 0,0002 |
|  | *L. infantum* | -0,89 | 1 x 10^-1^ | 34,2 [32,8 - 35,9] | 521,7 | 0,0004 |
| **18 S** | *L. amazonensis* | -1,74 | 1 x 10^-2^ | 35,8 [34,2 - 37,2] | 363,3 | 0,0003 |
|  | *L. braziliensis* | -1,87 | 1 x 10^-2^ | 35,6 [34,2 - 37] | 314,3 | 0,0003 |
|  | *L. guyanensis* | -1,95 | 1 x 10^-2^ | 34,9 [33,2 - 36,6] | 354 | 0,0003 |
|  | *L. panamensis* | -1,78 | 1 x 10^-2^ | 36 [34,2 - 37,7] | 323,7 | 0,0003 |
|  | *L. mexicana* | -1,84 | 1 x 10^-2^ | 35,7 [33 - 36,4] | 413,5 | 0,0003 |
|  | *L. infantum* | -1,87 | 1 x 10^-2^ | 35,8 [34,1 - 37,5] | 392,4 | 0,0003 |
