# Supplementary material for: Analytical Performance of Four Polymerase Chain Reaction (PCR) and Real Time PCR (qPCR) Assays for the Detection of Six Leishmania Species DNA in Colombia
Source: Front Microbiol. 2017 Oct 4;8:1907. doi: 10.3389/fmicb.2017.01907 (PMC5632848; doi:10.3389/fmicb.2017.01907)
Supplement: Supplementary file 5 [file Table_3.DOC]

**Table S3.** Post-hoc of targets _ Threshold cycle (Ct)

| **Target** | **Comparison** | **ARR** | | **LoD** | | **Accuracy** | |
| --- | --- | --- | --- | --- | --- | --- | --- |
|  |  | **Mean_diff** | **p-bonf** | **Mean_diff** | **p-bonf** | **Mean_diff** | **p-bonf** |
| **18S** | **HSP-70** | 0.106 | 1.000 | -0.095 | 0.210 | 4.161 | 0.000 |
|  | **ITS-1** | -1.022 | 0.000 | -0.473 | 0.000 | 0.621 | 0.000 |
|  | **kDNA** | 8.733 | 0.000 | 8.186 | 0.000 | 9.687 | 0.000 |
| **HSP-70** | **ITS-1** | -1-128 | 0.000 | -0.378 | 0.000 | -3.540 | 0.000 |
|  | **kDNA** | 8.677 | 0.000 | 8.281 | 0.000 | 5.525 | 0.000 |
| **ITS-1** | **kDNA** | 9.805 | 0.000 | 8.659 | 0.000 | 9.065 | 0.000 |

**Mean_diff:** mean differences, **p_bonf:** p-value of Bonferroni´s test.
